# Resectional One Anastomosis Gastric Bypass/Mini Gastric Bypass as a Novel Option for Revision of Restrictive Procedures: Preliminary Results

**DOI:** 10.1155/2018/4049136

**Published:** 2018-09-18

**Authors:** Roger Noun, Rita Slim, Ghassan Chakhtoura, Joseph Gharios, Elie Chouillard, Carla Tohmé-Noun

**Affiliations:** ^1^Department of Digestive Surgery, Hôtel-Dieu de France Hospital, University Saint Joseph Medical School, Beirut 166830, Lebanon; ^2^Dapartment of Gastroenterology, Hôtel-Dieu de France Hospital, University Saint Joseph Medical School, Beirut 166830, Lebanon; ^3^Department of Digestive Surgery, Poissy/Saint-Germain Medical Center, Université de Versailles/Saint-Quentin en Yvelines, Poissy, France; ^4^Department of Imaging, Clinique du Levant, University Saint Joseph Medical School, Beirut 50226, Lebanon

## Abstract

**Background:**

Revisional surgery is becoming a common and challenging practice in bariatric centers. The aim of this study was to evaluate resectional one anastomosis gastric bypass/mini gastric bypass (R-OAGB/MGB) as a revisional procedure.

**Methods:**

From January 2016 to February 2017, data on 21 consecutive patients undergoing R-OAGB/MGB for weight loss failure after primary restrictive procedures were prospectively collected and analysed.

**Results:**

Mean age was 39 ± 12 years (18–65), and 11 (52.3%) were women. The mean operative time was 96.4 ± 20.9 min (range, 122–80), and the mean postoperative stay was 47.8 ± 7.4 hours (range, 36–73). There were no deaths and no procedure-related complications. The mean body mass index (BMI) decreased from 42.9 ± 6.5 at the time of R-OAGB/MGB to 28.5 ± 4 at the 12-month follow-up. At that time point, the mean percentage of BMI loss (%EBL) and the mean percentage of total body weight loss (%TWL) reached 81.6 ± 0.17% and 35 ± 0.01%, respectively.

**Conclusion:**

R-OAGB/MGB was technically straightforward, effective, and safe in this at-surgical risk population. R-OAGB/MGB should be added to the armamentarium of revisional bariatric procedures considering its technical aspects and the potential advantage on weight loss.

## 1. Introduction

During the last decade, we have assisted to the disappearance of vertical banded gastroplasty and to progressive decline of laparoscopic gastric banding (LGB), while laparoscopic sleeve gastrectomies (LSGs) exponentially grew worldwide [1, 2]. Laparoscopic gastric plication (LGP) is an evolving technique that gained popularity with the idea of reproducing a sleeve gastrectomy but without tissue transection [3]. With an increasing number of purely restrictive procedures, the significant issue of weight regain is becoming more prevalent and many studies have reported unreliable long-term results [3–5].

Revision surgery represents today one of the main research fields in bariatric surgery and will most likely produce a major demand in the future and consequently, a significant economic burden [4–6].

Many surgical options are now available for revision, including those considered as an advancement and simplification of the standard biliopancreatic diversion by involving only a single anastomosis [7–9].

We report our preliminary experience with resectional one anastomosis gastric bypass/mini gastric bypass (R-OAGB/MGB), a procedure that starts by sleeve gastrectomy followed by an omega loop anastomosis between the transected sleeved gastric tube and the jejunum with special emphasis on operative and postoperative outcomes.

## 2. Materials and Methods

From January 2016 to February 2017, 21 patients (11 females, 10 males; age, 39.6 ± 12.2) who previously underwent primary restrictive procedures 10 LGB, 7 LSG, and 5 LGP were referred to our unit for weight regain. The mean BMI at the time of the primary procedure was 45 ± 4.8 kg/m^2^ and decreased to a nadir of 35 ± 5.3 and later increased to 42.9 ± 6.5 at the time of R-OAGB/MGB. None of the patients had previously undergone gastric band removal. In all patients, the previous procedure was performed by laparoscopy.

### 2.1. Preoperative Evaluation

**Figure 1:**
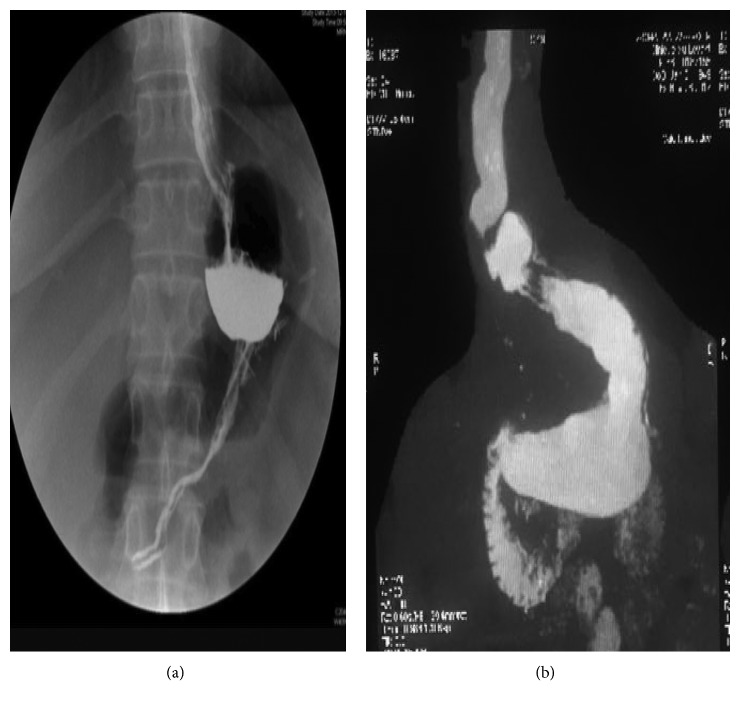
Group A: (a) dilated residual fundus; group B: (b) dilated remnant antrum.

**Figure 2:**
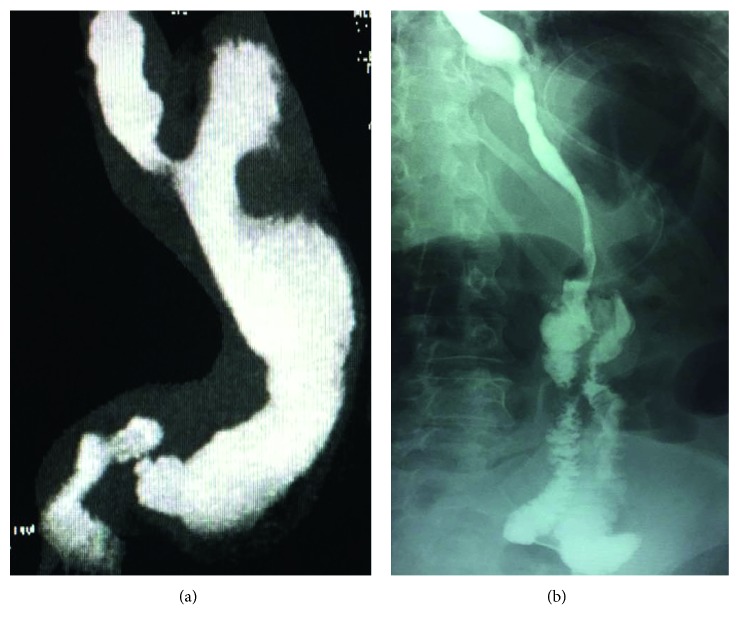
(a) Gastric prolapse following gastric plication; (b) postoperative X-ray swallow examination showing sleeve gastrectomy with omega loop anastomosis.

All patients were submitted to a preoperative anesthesiology workup including appropriate multidisciplinary counseling [10]. Patients with previous LGB were checked for gastric band erosion by preoperative gastroscopy, whereas the others received preoperative X-ray swallow to detect anatomical/surgical factors of weight regain. In patients with previous LSG, X-ray swallow examination detected 5 patients with dilated residual fundus (group A) and 2 patients with large remnant antrum (group B) (Figure 1). Patients with failed LSG and chronic symptoms of reflux were excluded from the current study. All patients with previous LGP had gastric prolapse of the gastric plication (Figure 2).

The risks, benefits, and long-term consequences of R-OAGB/MGB were discussed in detail during the initial encounter with the surgeon and the dietician. Written informed consent was obtained preoperatively from all patients. All patients received preoperative antibiotic prophylaxis and low-molecular-weight heparin.

### 2.2. Surgical Technique

The technique used for R-OAGB/MGB is based on a 5-port approach [11]. For patients with previous LSG, the gastric sleeve is dissected free from firm adhesion between the staple line and surrounding tissues, starting from the distal staple line and proceeding to the angle of His. For patients with previous LGP, the plicated part of the stomach was dissected free from surrounding tissues and the line of sutures was disrupted only where the first staples were placed. The first step of R-OAGB/MGB involved a calibrated (40 F tube) sleeve using 4.8 mm green Endo GIA reloads (Covidien, Boulder, CO) removing all the excessive and/or plicated gastric tissue along with plicature sutures.

For patients with previous LGB, the gastric band was freed from the surrounding capsule and adhesions and cut and extracted through the 15 mm port. The internal fibrous tissue between the band and the stomach was removed as well to prevent stenosis of the tube at this level. The gastric greater curvature was than completely freed starting at 4 cm proximal to the pylorus using LigaSure (Covidien, Minneapolis, MN, USA) along with the direct release of lower sac adhesions and scarring to the left crus. A sleeve gastrectomy was than performed as described above.

**Figure 3:**
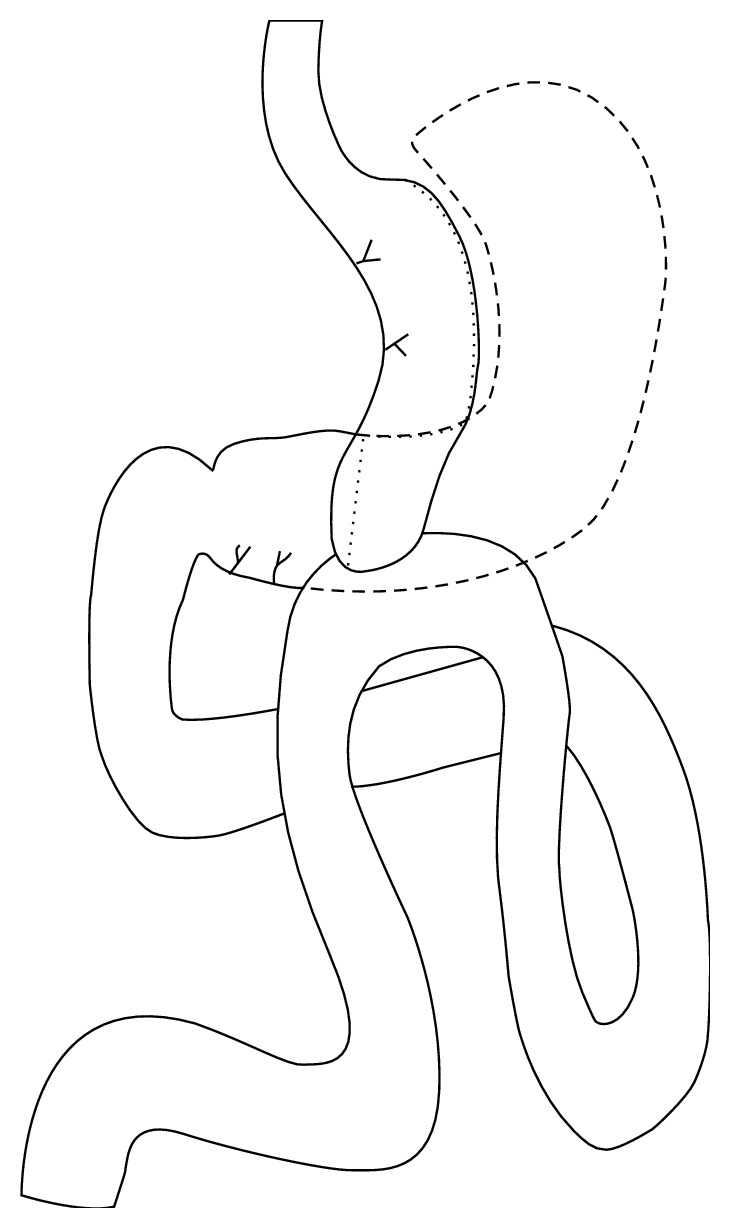
Drawing representing sleeve gastrectomy with omega loop anastomosis. The part in dotted line was removed.

The second step of R-OAGB/MGB involved transection of the sleeved tube at its base (at least 12 cm from the esophagogastric junction) and an antecolic loop end-to-side anastomosis with the jejunum (150 cm distal to the ligament of Treitz for patients with BMI ≤50 kg/m^2^ and 200 cm for patients with BMI >50 kg/m^2^) (Figure 3). Also, we used a hanging suture between the gastric pouch and the afferent loop to minimize reflux and a retaining suture between the lower part of the pouch and the antrum to prevent it from twisting.

Intraoperative methylene blue test was performed to exclude a leak. Increasing systolic blood pressure to 130 mm Hg while decreasing the pneumoperitoneum pressure allowed the achievement of hemostasis at the staple line by cautery or oversuturing. The specimens were retrieved from the 15 mm port. No abdominal drainage was left in place. Every patient who underwent a bariatric operation in our division had a DVD recorded video from the laparoscopic camera, that allows for time recording and video staff presentations, as well as a reference in case of medicolegal issues.

### 2.3. Postoperative Care

All patients were strongly instructed for early postoperative ambulation and were allowed to start drinking water on day one postoperatively. Upon discharge, all the patients got detailed dietary instruction sheet and were instructed to take supplemental minerals, multivitamins, and proton pump inhibitor for at least 6 months. Follow-up appointments were scheduled through a calendar sheet. Patient contact with a surgeon and dietitian was guaranteed through phones numbers or online.

### 2.4. Endpoints

The primary endpoints included intraoperative data (intraoperative complications, operative time, and conversion) and postoperative outcome (30-day mortality or morbidity and length of hospital stay). Follow-up data included weight loss parameters and evolution of comorbidities. Remission of type 2 diabetes was defined as the fasting plasma glucose level <126 mg/dL and HbA1c level <6.5% requiring no medications [12]. Remission of dyslipidemia and hypertension was defined as the normal lipid panel and blood pressure <135/85 mmHg without medication. Remission of sleep apnea syndrome was considered when stopping continuous positive airway pressure or absence of symptoms strongly suggesting sleep apnea. Partial improvement was considered when considering the number or dosage of the drugs used for the treatment of comorbidities or partial regression of symptoms.

The percentage of excess BMI loss (%EBL) is calculated by dividing the change in BMI from the baseline by excess BMI which corresponds to the initial BMI minus the ideal BMI (25 kg/m^2^).

Data analysis was carried out using the SPSS software version 21. Results are reported as mean ± SD or as percentages when appropriate.

## 3. Results

All procedures were completed laparoscopically and were uneventful. None were admitted to the intensive care unit. The mean operative time as recorded by the camera was 96.4 ± 20.9 min (range, 122–80), and the mean postoperative stay was 47.8 ± 7.4 hours (range, 36–73). There were no deaths and no procedure-related complications. One patient with preoperative gastric prolapse following gastric plication underwent postoperative X-ray swallow examination for epigastric pain showing unremarkable sleeved bypass with the long and narrow gastric tube. None of the patients complained of symptoms of chronic reflux or bile regurgitation.

**Figure 4:**
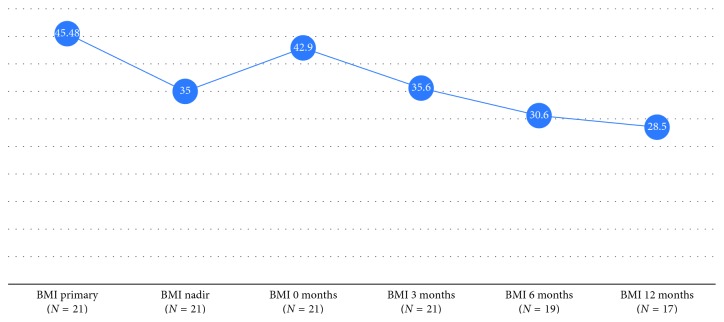
Evolution of body mass index (BMI) from the primary operation through the resectional MGB.

The mean BMI decreases to 35.6 ± 5.6, 30.6 ± 4.6, and 28.5 ± 4 at 3, 6, and 12 months of follow-up, respectively. At that time points, the mean %EBL reached 41.7 ± 0.1, 73.7 ± 0.1, and 81.6 ± 0.17%, while the mean percentage of the total body weight loss (%TWL) reached 17 ± 0.01, 29.2 ± 0.01, and 35 ± 0.01% (Figure 4). Three patients with previous LGB had complete resolution of diabetes, and two with previous LGP had complete resolution of hypertension.

## 4. Discussion

Weight loss failure after bariatric procedures remains problematic with regard to its surgical management. Revisional surgery is becoming a common practice in bariatric centers. Today, as many as 15% of bariatric procedures are revisional, and this number is prone to an increase in upcoming years [13, 14]. Revision procedures are often technically challenging for surgeons due to altered anatomy and to firm adhesions following the primary procedure. Revisional surgery has been associated with increased perioperative surgical complications arising from the gastric pouch or from the gastric remnant [8, 10, 14].

LSG is a perfect concept of a simple and effective operation and is currently the leading bariatric procedure worldwide. Now that long-term data are being reported, it is evident that weight loss failure following LSG is significant with a conversion rate of up to 35.8% at ten years [15–17]. LGP is an evolving bariatric procedure, unfortunately hampered by a high surgical revision rate reaching 57.7% at 18 months [3, 6, 18]. Anatomical/surgical factors of weight regain after LSG includes an initial large sleeve, incompletely resected fundus, and a large remnant antrum, whereas those after LGP includes dilatation or gastric prolapse of the gastric plication as observed in the current series [5, 18]. In this setting, revisional LSG is the most obvious option for both procedures, but stapling of scarred and thickened tissues may lead to an increased risk of leakage [17, 19]. Also, the LSG pouch is prone to reenlargement with time and may require a second salvage procedure and additional costs.

Single anastomosis duodenoileal bypass with sleeve gastrectomy (SADI-S) is considered today as an effective salvage procedure. However, it is burdened by the risks of duodenal fistula and malnutrition [20]. Alternatively, conversion to a functional single-anastomosis gastric bypass is a recent option for revision. However, this procedure is prone to device-related complications and to malnutrition in patients necessitating long loops (large gastric pouch) [21].

We have previously reported excellent results of primary OAGB/MGB and revisional OAGB/MGB in terms of efficiency, safety, and weight maintenance because of its balanced restrictive-malabsorbtive effects [8, 22]. Compared to OAGB/MGB, R-OAGB/MGB is a technical modification that facilitates the pouch fashioning, allows direct access to adhesions of the lesser sac, avoids complications arising from the residual stomach, and might have metabolic implication. It first starts by a sleeve gastrectomy rather than fashioning of an OAGB/MGB pouch and is therefore much easier to perform [23]. Also, the advantage of resection allowed direct access to severe adherences and scarring induced by the primary procedure that represents hazardous steps during revision. This technical ease translated into a shorter operative time as compared to other revisional OAGB/MGB series [8, 10, 24]. The second step is similar to OAGB/MGB by performing an omega loop anastomosis rendering the stress on the thickened gastric wall minimal, thus eliminating the risk of leakage that did not occur in the present series. Also, postoperative complications including bleeding, acute dilatation, and leaks, arising from the remnant stomach, were also eliminated while postoperative hospital stay compares favorably with others [8, 10, 24–27]. In the long run, the risk of cancer arising from the resected stomach with an estimated incidence of 0.03% is also discarded [27–31]. Notably, EBL and TWL at one year after R-OAGB/MGB exceeded those reported after revisional OAGB/MGB series [8, 13, 24, 32] and even after some primary OAGB/MGB series [22, 33, 34]. The rationale for this final result could be related to calibration and to possible metabolic effects of fundectomy [29, 35, 36]. However, comparative studies are necessary before drawing any conclusion concerning this final result.

## 5. Conclusion

Reflecting the study's results, the procedure described herein was technically straightforward, effective, and safe in this at-surgical risk population. We believe that this technical modification facilitates the pouch fashioning considering the vast majority of bariatric surgeons who are familiar with sleeve gastrectomy. R-OAGB/MGB should be added as a viable option to the armamentarium of revisional bariatric procedures and may be proposed in the future as a primary bariatric procedure considering its technical aspects and the potential advantage on weight loss. However, comparative studies are needed to confirm this last issue.

## Data Availability

The data used to support the findings of this study are available from the corresponding author upon request.
